# Ocular Manifestations in Congenital Insensitivity to Pain with Anhidrosis: A Window into a Rare Syndrome

**DOI:** 10.3390/vision9030062

**Published:** 2025-07-21

**Authors:** Mohammed Baker, Kenda Abedal-Kareem, Sadeen Eid, Mahmoud Alkhawaldeh, Yahya Albashaireh, Jihan Joulani, Sara Bani Amer, Ethar Hazaimeh, Omar F. Jbarah, Abdelwahab Aleshawi, Rami Al-Dwairi

**Affiliations:** 1 Faculty of Medicine, Jordan University of Science and Technology, Irbid 22110, Jordan; mbbaker19@med.just.edu.jo (M.B.); kyabedalkareem19@med.just.edu.jo (K.A.-K.); sseid19@med.just.edu.jo (S.E.); mhalkhawaldeh19@med.just.edu.jo (M.A.); yzalbashaireh19@med.just.edu.jo (Y.A.); jojoulani19@med.just.edu.jo (J.J.); ssbaniamer19@med.just.edu.jo (S.B.A.); 2 Medical Researcher, Neuropedia for Training and Scientific Research, Amman 11194, Jordan; ethar.haz92@gmail.com (E.H.); o.jbarah@gmail.com (O.F.J.); 3 Department of Neurology and Cerebrovascular Medicine, Saitama Medical University International Medical Center, Hidaka, Saitama 320-1298, Japan; 4 Department of Cerebrovascular Surgery, Saitama Medical University International Medical Center, Hidaka, Saitama 320-1298, Japan; 5 Division of Ophthalmology, Department of Special Surgery, Faculty of Medicine, Jordan University of Science and Technology, P.O. Box 3030, Irbid 22110, Jordan; abdelwahhabjamal@yahoo.com

**Keywords:** CIPA, corneal ulcer, NTRK1 gene, PRISMA

## Abstract

**Background**: Congenital insensitivity to pain with anhidrosis (CIPA) is a rare autosomal recessive syndrome caused by loss-of-function mutations in the Neurotrophic Tyrosine Kinase Receptor 1 gene, characterized by recurrent episodes of infections and unexplained fever, anhidrosis, absence of reactions to noxious stimuli, intellectual disability, self-mutilating behaviors, and damage to many body organs, including the eyes. **Main text**: We systematically searched the Medline/PubMed, Scopus, and Web of Science databases from their inception until March 2025 for papers describing the clinical manifestations of patients with CIPA. The inclusion criterion was papers reporting ocular manifestations of patients diagnosed with CIPA. We excluded non-English papers or those reporting ocular manifestations of patients diagnosed with syndromes other than CIPA. Also, we excluded review articles, clinical trials, gray literature, or any paper that did not report ocular manifestations of patients with CIPA or that reported patients with previous ocular surgeries. Out of 6243 studies, 28 were included in the final analysis, comprising 118 patients. The mean age was 7.37 years, and males represented 63.5% (n = 75). Of the patients, fifty-six had bilateral ocular manifestations. The most common ocular manifestations were the absence of corneal reflex in 56 patients (47.5%, bilateral in 56), whereas corneal ulcerations were the second most common manifestation in 46 patients (38.98%, bilateral in 8), followed by corneal opacity in 32 patients (27.11%, bilateral in 19). Topical lubricants, topical antibiotics, and lateral tarsorrhaphy were common management modalities for these patients. Absent corneal sensitivity, corneal ulcers, and corneal opacities, among other manifestations, are common ocular presentations in patients with CIPA. **Conclusions**: Self-mutilation, intellectual disability, decreased lacrimation, and absence of the corneal reflex are factors that may explain the development of these manifestations in CIPA. The early detection of these manifestations can improve patient conditions and prevent further complications, in addition to helping to guide the clinical diagnosis of CIPA in these patients.

## 1. Introduction

Congenital insensitivity to pain with anhidrosis (CIPA), also known as hereditary sensory and autonomic neuropathy type IV (HSAN-IV), is a rare autosomal recessive neuropathy marked by the inability to detect noxious stimuli such as pain and heat [1]. The exact incidence of this disorder remains unclear, but CIPA is very widespread in Japanese and Israeli Bedouin populations; Hage et al. estimated a prevalence of 1 in 600,000–950,000 live births a year in Japan [2]. Individuals diagnosed with CIPA are prone to self-harm, which can result in severe and lifelong impairments [1]. The signs and symptoms of CIPA appear early and usually present in infancy as unexplained episodes of hyperpyrexia. However, with careful medical attention, affected individuals can live into adulthood [2].

CIPA is caused by mutations in the Neurotrophic Tyrosine Kinase Receptor 1 gene (NTRK1), which impair the survival of nociceptive sensory neurons and sympathetic ganglion neurons that are reliant on nerve growth factor (NGF) [3]. Neuropathological studies indicate deficiencies in the dorsal root ganglia (DRG), characterized by a lack of development of small nociceptive neurons, a severe loss of unmyelinated and small-diameter nerve fibers in afferent neurons, and abnormal innervation of eccrine sweat glands by cholinergic sympathetic fibers [4,5]. The impaired innervation leads to a reduced or absent pain sensation against traumatic insults, in addition to the loss of normal sweating in response to elevated body temperatures.

Accordingly, CIPA is characterized by decreased pain sensitivity, anhidrosis, and intellectual disability, and patients may experience a series of painless injuries that result in joint abnormalities, chronic osteomyelitis, burns, and fractures [6]. Self-mutilation is frequent and includes biting the tongue, lips, and fingers [6]. These people suffer anhidrosis, which causes episodes of fever of unknown origin and can lead to death from hyperpyrexia [7,8]. Although intellectual disability is a common finding, the exact pathophysiologic cause of this condition is unclear [9].

The ocular symptoms of CIPA include dry eye syndrome, superficial punctate keratitis, corneal opacity, neurotrophic keratopathy, and corneal ulcers due to decreased corneal innervation resulting from the loss of NGF-dependent neurons [10,11]. The absence of corneal sensitivity, which causes corneal ulcers and opacities, is one of the visual symptoms of this illness [12]. Neurotrophic keratitis (NK) is caused by CIPA and is associated with altered neurotransmitter levels. Reduced corneal sensation is a defining feature of NK cells [13].

Although multiple reports describing ocular findings of patients with CIPA worldwide have been published, no single literature review gathering and organizing these findings and their associations has been conducted, which leaves a gap in our understanding of the long-term effects and contributing variables. To fill this gap, our systematic review provides a comprehensive approach to ocular involvement in CIPA by investigating a variety of topics, including the most common ocular findings in patients with CIPA, the main genetic abnormalities, the possible correlation between age and sex and the distribution of ocular involvement, an analysis of the interventions these patients received, and the frequency of consanguinity in their family history.

## 2. Patients and Methods

### 2.1. Protocol and Registration

This systematic review is reported according to the Preferred Reporting Items for Systematic Reviews and Meta-Analyses (PRISMA) statement [14] and was registered in the Prospero online database (protocol ID: CRD42023389034).

### 2.2. Search Strategy and Information Sources

One reviewer constructed the search strategy by combining the terms “congenital insensitivity to pain with anhidrosis” and “hereditary sensory and autonomic neuropathy type IV” and their synonyms. The same reviewer systematically searched the PubMed/Medline, Scopus, and Web of Science databases from their inception until March 2025. We also performed backward and forward reference searches of the reference lists of the included studies. No language restriction was made during the search process. The detailed search strategy is as follows:

#### 2.2.1. Medline/PUBMED

(CIPA [Title/Abstract]) OR (Congenital insensitivity to pain with anhidrosis [Title/Abstract]) OR (congenital indifference to pain with anhidrosis [Title/Abstract]) OR (HSAN IV [Title/Abstract]) OR (HSAN type 4 [Title/Abstract]) OR (“Hereditary sensory and autonomic neuropathy IV” [Title/Abstract]) OR (“Hereditary sensory and autonomic neuropathy type 4” [Title/Abstract])

#### 2.2.2. Scopus

(TITLE-ABS(CIPA)) OR (TITLE-ABS(“Congenital insensitivity to pain with anhidrosis”)) OR (TITLE-ABS(“congenital indifference to pain with anhidrosis”)) OR (TITLE-ABS(“HSAN IV”)) OR (TITLE-ABS(“HSAN type 4”)) OR (TITLE-ABS(“Hereditary sensory and autonomic neuropathy IV”)) OR (TITLE-ABS(“Hereditary sensory and autonomic neuropathy type 4”))

#### 2.2.3. Web of Science

((TI = CIPA OR AB = CIPA)) OR ((TI = “Congenital insensitivity to pain with anhidrosis” OR AB = “Congenital insensitivity to pain with anhidrosis”)) OR ((TI = “congenital indifference to pain with anhidrosis” OR AB = “congenital indifference to pain with anhidrosis”)) OR ((TI = “HSAN IV” OR AB = “HSAN IV”)) OR ((TI = “HSAN type 4” OR AB = “HSAN type 4”)) OR ((TI = “Hereditary sensory and autonomic neuropathy IV” OR AB = “Hereditary sensory and autonomic neuropathy IV”)) OR ((TI = “Hereditary sensory and autonomic neuropathy type 4” OR AB = “Hereditary sensory and autonomic neuropathy type 4”))

### 2.3. Eligibility Criteria

The inclusion criteria for patients were as follows: (1) diagnosed with congenital insensitivity to pain with anhidrosis (CIPA); (2) case description mentions ocular manifestations of patients diagnosed with CIPA. Exclusion criteria were as follows: (1) patients diagnosed with diseases other than CIPA (including congenital insensitivity to pain); (2) non-English papers; (3) no mention of ocular manifestations of patients diagnosed with CIPA; (4) papers lacking full text; (5) review articles, randomized controlled trials, cross-sectional surveys, letters to the editor, correspondence, or animal studies; (6) gray literature (such as conference abstracts, theses, etc.); and (7) papers reporting patients with CIPA with a history of ocular surgery prior to their presentation.

### 2.4. Study Selection and Screening

Two reviewers independently screened the titles and abstracts of the studies to determine whether they met the inclusion criteria using the “Rayyan” website after deduplication. The reviewers subsequently evaluated the full texts of the included studies from the title/abstract phase and excluded those studies that did not meet the inclusion criteria. Any dispute between the two reviewers was resolved through a meeting with a third reviewer.

### 2.5. Data Extraction

Six authors extracted data from the included studies using a preformed extraction tool, which is an Excel sheet file that the authors made based on their discussion and according to the review objectives and target outcomes, without any AI assistance. The authors extracted the following data: (1) patient demographics and study characteristics; (2) ocular manifestations of each patient and eye involvement; (3) investigations, interventions, and outcomes; (4) risk factors suggested to contribute to ocular manifestations (such as self-mutilation and intellectual disability); and (5) genetic defects, family history of inherited disease, and family history of consanguinity. The seventh author reviewed all the data to unify the terms used in the data extraction.

### 2.6. Quality Assessment of Studies

Two independent authors assessed the quality of the included studies using The Joanna Briggs Institute (JBI) Critical Appraisal Checklist for Case Reports [15]. Studies were rated out of 8 points as low (0–2 points), intermediate (3–6 points), or high (7–8) quality.

### 2.7. Data Analysis

Given the descriptive nature of this review, we used descriptive statistics to present the data. Categorical and dichotomous variables are presented as frequencies and percentages, whereas continuous variables are presented as the mean and standard deviation, where possible.

## 3. Results

### 3.1. Study Characteristics and Patient Demographics

**Figure 1:**
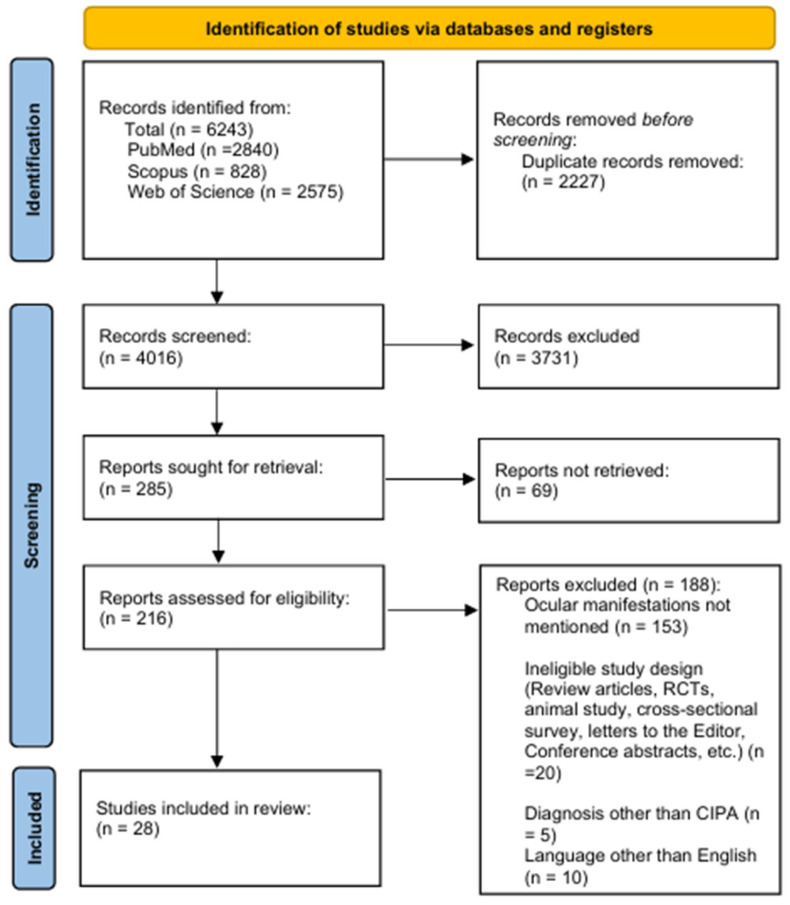
PRISMA flow diagram of the included studies.

A PRISMA flow diagram of the selected articles is shown in Figure 1. A total of 6243 records were identified, and 2227 duplicates were removed, leaving 4016 records. A total of 3731 records were excluded after title/abstract screening, leaving 285 records for full-text screening, of which 69 were available as abstracts only. After the exclusion of 188 records that did not meet the inclusion criteria, a total of 28 articles, comprising 118 individual patients from 14 countries, were included in this review (Table 1). We checked and ensured that the data for each patient was included only once in the final analysis.

In terms of quality assessment, 13 studies were of high quality, whereas 15 studies were of intermediate quality. No study scored as low quality (see Supplementary File S1).

The largest proportion of the included studies were from the Middle East (n = 12). Males comprised 63.6% (n = 75), and the mean age was 7.37 years. Eighty-seven patients had self-mutilating behaviors (73.7%), whereas 93 (78.8%) patients had an intellectual disability.

### 3.2. Ocular Manifestations

Table 2 summarizes the distribution of ocular manifestations. A total of 20 different ocular manifestations were identified in this review, of which absent corneal sensitivity was the most common manifestation (47.5%, n = 56). Corneal ulcerations were the second most common manifestation (39.83%, n = 47), followed by corneal opacity (n = 33, 27.96%). Fifty-six patients had at least one bilateral manifestation (47.45%), and 100% of patients with absent corneal reflex had bilateral involvement, whereas 8 and 19 of those with corneal ulceration and corneal opacity, respectively, had bilateral involvement.

#### 3.2.1. Common Ocular Manifestations

Twenty-seven studies reported the five most common manifestations in patients with CIPA: absence of corneal reflex (47.5%, n = 56), corneal ulceration (40%, n = 46), corneal opacity (27.96%, n = 33), decreased lacrimation (22%, n = 26), and superficial punctate keratopathy (16.1%, n = 19). Shatzky et al. reported 12 (10.2%) patients with corneal ulceration and preserved corneal sensitivity [22]. Elsana et al. reviewed 32 (27.1%) patients with CIPA, 8 (2.5%) of whom had corneal ulceration and 10 (8.5%) of whom had corneal opacities; these patients were suffering from self-mutilation behaviors, a suspected risk factor for ulcers, which are more common in children younger than 10 years old [10]. In addition, 9 (7.6%) patients had decreased lacrimation and 13 (40.6%) had an absent corneal reflex [10]. Yagev et al. reported 8 (6.8%) children with corneal ulcers and 10 (8.5%) patients with corneal opacities [21]. Jarade et al. [23] and López-Cortés et al. [40] each reported one patient (1.7%) with bilateral corneal ulceration. Masri et al. reported four (3.3%) patients with corneal ulceration from Jordan [39], whereas Shorer et al. reported seven (6%) patients with corneal ulcers [24]. Levy et al. [26], Gao et al. [30], Rapp et al. [31], Ofluoglo et al. [34], Schalka et al. [27], and Suresh et al. [38] each reported a single patient with CIPA (5%) with corneal ulceration. Amano et al. reported 18 (15.2%) patients with CIPA, 12 (10.2%) of whom had superficial punctate keratitis, 2 (1.6%) had corneal opacities, 7 (6%) had decreased lacrimation, and 6 (5%) lacked corneal sensation [28]. Bilateral opacities were reported in four (3.4%) patients by Altassan et al. [36], Guven et al. [33], Brahim et al. [18], and Kucukdurmaz et al. [29]. Vardy et al. reported a patient (0.8%) with bilateral opacities in addition to decreased lacrimation, absent corneal sensitivity, and self-mutilating behaviors [16]. In addition to Amano et al. [28] and Elsana et al. [10], Sethi et al. reported a patient with CIPA (0.8%) with superficial punctate keratitis with decreased lacrimation and self-mutilation behavior and was found to have neurotrophic keratitis [41].

Regarding geographical distribution, we found that some manifestations tend to be more common in certain countries. Absent corneal reflex was notably reported in Israel (n = 29, 51.8%) and, to a lesser extent, in Japan (n = 6, 10.7%) and China (n = 5, 8.9%). The same applies to decreased lacrimation (Israel: n = 10 (38.5%), Japan: n = 7 (26.9%)), corneal ulceration (Israel: n = 35, 76%), and corneal opacity (Israel: n = 22, 68.7%). However, superficial punctate keratopathy was reported more in Japan (n = 12, 63.1%) than in Israel (n = 6, 31.6%).

#### 3.2.2. Rare Ocular Manifestations

Rare manifestations identified in this review were reported by 14 studies, which included blepharoptosis, neurotrophic keratitis, exotropia, esotropia, corneal scarring, eye redness, ciliary entropion, keratoconus, epiphora, astigmatism, congenital keratitis, hypopyon, sluggishly reactive pupil, blurred vision, and tortuous retinal arteries (Table 2). Among them, blepharoptosis was the most common (n = 8, 6.7%) and was bilateral in one patient, followed by neurotrophic keratitis (n = 4, 3.3%), exotropia (n = 4, 3.3%), esotropia (n = 4, 3.3%), and corneal scarring (n = 4, 3.3%).

### 3.3. Potential Medical Treatments of Ocular Abnormalities and Their Outcomes

Only eight papers reported an intervention for their patients. Jarade et al. treated a patient with corneal ulcers via frequent installation of preservative-free artificial tears in both eyes, using soft therapeutic bandage contact lenses and topical antibiotics to prevent bacterial superinfection [23], leading to complete ulcer healing. Levy et al. treated a patient with large corneal erosion using protective tarsorrhaphy, which resulted in complete resolution of the erosion [26]. In addition, Guven et al. treated a patient with extensive bilateral corneal opacity using lateral tarsorrhaphy [33]. The same applies to Yagev et al., who surgically treated five patients with corneal ulceration (lateral tarsorrhaphy in five patients, two patients (one eye each) had corneal patch grafts, and one patient (one eye) had a penetrating keratoplasty) [21]. John et al. treated their patient with lubricants in both eyes and topical antibiotics in the right eye. She underwent bilateral punctal cautery of both the upper and lower puncta [13]. After 1 year, in addition to topical lubricants, she was treated with topical antibiotics and underwent bilateral lateral tarsorrhaphy for corneal erosion [13]. In the past 10 years, she has had recurrent corneal lesions requiring inpatient and outpatient care, with topical antibiotics and lubricants [13]. However, punctate erosions persist despite treatment. Elsana et al. used topical fortified antibiotics (ceftazidime and vancomycin) for corneal ulceration in all patients, photoactivated chromophores for keratitis in one patient with good ulcer resolution, lubricating eyedrops for patients with dry eyes, and surgical interventions, such as lateral tarsorrhaphy and amniotic membrane transplantation, for the treatment of nonhealing corneal ulcers in four eyes of patients with CIPA [10]. Verity et al. injected 4% cocaine and 1% hydroxyamphetamine into the left eye of a patient, which failed to produce mydriasis or relieve ptosis; however, drops containing 0.5 percent cyclopentolate and 5 percent phenylephrine dilated the pupils and relieved ptosis [17]. Suresh et al. treated neurotrophic keratitis with 0.3% tobramycin eye drops three times per hour in addition to atropine eye drops and 0.5% carboxymethyl cellulose eye drops for a week [38]. In contrast, they treated corneal ulceration with bilateral lateral tarsorrhaphy [38].

The outcomes reported in most of these studies show that medical treatment can be considered as an effective first-line therapy for managing critical lesions such as corneal ulceration and corneal opacity while considering surgical procedures, such as lateral tarsorrhaphy and penetrating keratoplasty, as a definitive treatment for recurrent nonhealing lesions.

### 3.4. Genetic Defects and Family History of Inherited Diseases

Twenty-seven different mutations in the NTRK-1 gene were reported in the included studies. López-Cortés et al. reported the homozygous mutant genotype T/T of the missense mutation rs80356677 (Asp674Tyr), whereas the pathogenic mutation was rs763758904 (Arg602*) [40]. Masri et al. identified the mutation c. 1860_1861insT; p.Pro621fs in four patients from Jordan [39]. Gao et al. reported compound heterozygote mutations, namely, c.1561T-C in exon 13 and c.2057 G-A in exon 15, inherited from each parent, which predict the amino acid substitutions p.F521L (phenylalanine acid—R-leucine) and p.R686H (arginine—R-histidine) [30]. Shatzky et al. identified “TrkA: 1926-ins-T” and “TrkA: Pro-689-Leu” mutations in one family [22], whereas Shorer et al. found the same 1926-ins-T mutation in the TrkA gene (24), which was identified in 13 patients by Elsana et al. [10]. Altassan et al. identified three missense mutations (p.Arg110Asp, p.Arg643Gln, p.Leu694- Pro) and two nonsense mutations (p.Ser146Ter, p.Lys476Ter) [36]. Geng et al. reported five missense mutations (c.1784T>G, c.1927C>T, c.2056C>T, c.2152G>A, and c.2293C>T), resulting in amino acid changes (L595R, R643 W, R686C, G718S, and R765C, respectively); one nonsense mutation (c.1786C>T, R596*); two frameshift mutations (c.963delG, c.1736delT); and four intronic splicing mutations (c.851-33T>A, c.287+2dupT c.850+1G>A, c.2188-11G>A) in five patients from China [37]. Guven et al. reported a homozygous c.2001C-T alteration in exon 15 in both twins [33]. Rapp et al. reported the homozygous c.274dupG, pGlu92GlysfsX81 mutation in the NTRK1 gene on chromosome 1 [31]. Hiura et al. reported a homozygous deletion of a single base “C” at nucleotide 1726 in exon 14 of the tropomyosin receptor kinase A (TRKA) gene [25]. Suresh et al. reported that their Indian patient was homozygous for the pathogenic frameshift mutation NTRK1 c.717delG and pMet239fs, and the parents were heterozygous for the same mutation [38]. Table 3 shows the association between reported mutations and corresponding ocular manifestations. We only observed an association between corneal ulcerations and the “TrkA: 1926-ins-T” mutation, which was found in 20 cases (43.47%).

In relation to inherited diseases, several patients had a family history of similar conditions or other inherited diseases, and 64 patients (54.23%) had a family history of consanguinity. A patient described by Jarade et al. had a cousin with a similar clinical picture [23]. Masri et al. reported that of their four patients, two had one brother with CIPA, one had one sister with CIPA, and one had one uncle with CIPA, who died of chronic renal failure [39]. Guven et al. reported twins with CIPA [33]. Vardy et al. reported that their patient had a brother with ataxia telangiectasia and a sister with CIPA [16]. Iftikhar et al. reported a history of infant death due to recurrent high-grade fever in six of their patient’s siblings [32]. Verity et al. reported that the patient’s mother was hypothyroid, his father had a genotype sensitive to succinylcholine and had developed a teratoma of one testis, and his brother died suddenly of crib death at five months of age [17]. Finally, Othman et al. reported that their patient had four siblings with similar conditions [35].

## 4. Discussion

Congenital insensitivity to pain with anhidrosis (CIPA), also known as hereditary sensory and autonomic neuropathy type IV (HSAN IV), is an autosomal recessive disorder characterized by recurrent episodic fevers, anhidrosis, absence of reaction to noxious stimuli, nonhealing ulcers, intellectual disability, and self-mutilating behavior, especially involving the fingertips, tongue, and cornea [38]. Neuropathological studies have shown that CIPA is caused by mutations in NTRK1 that result in autophosphorylation in response to nerve growth factor (NGF) [3]. Our systematic review appears to be the first attempt to determine the distribution of ocular manifestations in patients with CIPA.

Although we remain cautious in making definitive conclusions, there is consistency in the types of health complications that occur in patients with CIPA. Our systematic review showed that the most common manifestations were absent corneal sensitivity in 56 patients (47.5%), followed by corneal ulcerations (39.8%, n = 47). The third most common ocular manifestation was corneal opacity in 33 patients (27.96%), followed by superficial punctate keratopathy in 19 patients (16.1%). However, 56 patients were diagnosed with CIPA and had at least one bilateral ocular manifestation. Corneal denervation or a partial decrease in corneal innervation causes different types of corneal diseases and is likely one of the causes of the suppression of tear secretion and mucin expression, which explains the pathology of the corneal epithelium [28].

Interestingly, we found a link between corneal ulcerations and the mutation “TrkA: 1926-ins-T”, which was found in 20 cases (43.47%). This may reflect certain pathological processes on the molecular level that contribute to corneal pathologies. Also, this provides a valuable guide to predicting certain ocular lesions and improving the timely detection of ocular lesions in patients at risk, thereby avoiding further complications. Although we were unable to find a link to other manifestations, we recommend that further analytical studies be conducted to determine if certain mutations can be considered as genetic markers for certain manifestations.

There are common treatment approaches available for most patients with ocular manifestations, such as topical lubricants, topical antibiotics, and lateral tarsorrhaphy. Amano et al. and Indo et al. suggested that these patients will benefit from routine care for dry eyes, prevention of corneal infection, and daily observation of the ocular surface to maintain good visual function [28,42]. Therefore, all patients must consider daily care for both eyes as well as ophthalmologic examination and follow-up.

However, although limited, the reported data from the included studies showed that medical treatment can be considered as the first choice when managing vision-threatening lesions like corneal ulcerations and opacities, while surgical interventions such as lateral tarsorrhaphy were commonly more effective in treating such pathologies, especially for recurrent and nonhealing lesions. The absence of a reliable management approach for ocular pathologies in patients with CIPA highlights the importance of establishing standardized management guidelines to improve decision-making when dealing with such patients.

On the other hand, patients with CIPA had not only ocular manifestations but also specific common symptoms. Among the patients in our review, there were 83 with self-mutilation (70.3%), and intellectual disability occurred in 71 patients (60.1%). Amano et al. suggested that oral self-mutilation might be induced in children with HSAN type IV through excessive bruxism or autoextraction in response to malaise or discomfort accompanying tooth eruption [28]. The vestigial presence of sensitivity to pain in selected areas that was observed in all patients may suggest that parts of the body, including certain nerves, are only partially affected by the pathological process, which could be caused by incomplete expression of the gene or by an escape phenomenon or be the result of nonspecific TrkA receptor (p75 NGFR) activity creating alternative sensory pathways [43].

Although this disorder is rare, the findings in this review reinforce the need for training in the recognition of symptoms of CIPA for patient care. Awareness of its characteristic features may lead to earlier diagnosis and prenatal diagnostic decisions in future pregnancies. Findings of a high occurrence of certain ocular irregularities among patients of different backgrounds are encouraging. Physicians and parental knowledge about associated complications may offer patients a good chance for a better lifestyle. In addition, the family history of the patient should be taken into consideration for possible genetic inheritance patterns.

This review has several limitations. As this was a systematic review, most of the available studies were case reports. Additionally, in our study selection process, we excluded papers written in non-English languages. This may affect the generalizability of our results, as some findings reported in these studies may reflect geographical variation in the distribution of certain ocular manifestations. Moreover, most of the included studies were from Asia, which limits the generalizability of our findings outside the mentioned countries due to genetic variations and differences in social behaviors regarding familial disease inheritance. More reports on ocular manifestations among patients with CIPA are expected to be found; these patients were not included in this systematic review due to the rarity of the disorder in the general population and possible poor documentation in some areas. Furthermore, studies on a larger series of patients with CIPA are needed to confirm the differences in severity between the entities and to elucidate their mechanism, and further analysis is required to confirm the precise effect of this mutation on the transcript.

As for future research, we recommend establishing international registries for patients with CIPA to improve international collaborative studies to deepen our understanding of the disease mechanism. Also, we encourage genetic studies to be conducted in these patients to help find genetic biomarkers for diagnosis and follow-up, which will contribute to the discovery of genetic therapies based on identified molecular targets.

## 5. Conclusions

In this review, we found that the absence of corneal sensitivity, corneal ulcers, corneal opacities, and superficial punctate keratopathy, among other manifestations, are common ocular presentations in patients with CIPA. Self-mutilation, intellectual disability, decreased lacrimation, and absence of the corneal reflex are factors that may explain the development of these manifestations. The proper detection of these manifestations can improve patient conditions and prevent further complications, in addition to helping to guide the clinical diagnosis of CIPA in these patients.

## Figures and Tables

**Table 1:** Characteristics of the included studies.

Authors	Sample Size (Cases)	^a^Age (Years)	Sex		Country	Study Design	Ocular Manifestations	Ophthalmic Examinations/Investigations	Comorbidities
			Female	Male					
Vardy et al., 1979 [16]	1	0.33	1	0	Israel	Case report	Corneal opacities	N/A	Hypotonia, hypersalivation, osteomyelitis
Verity et al., 1982 [17]	1	4.00	0	1	UK	Case report	blepharoptosis, retinal arteries were unusually tortuous	N/A	Bilateral Horner’s syndrome, osteomyelitis, fractured lumbar verte bra, poor temperature control, ab normal behavior, left otitis media, rupture of the eardrum, self-mutilated fingers, diminished sensitivity to pain
Brahim et al., 1987 [18]	2	9.00	0	2	USA	Case report	Corneal opacities	N/A	Dry skin, shortened fingertips scarred by trauma, absence of sensation to superficial, deep, or visceral pain
Kashtan et al., 1992 [19]	1	23.00	1	0	USA	Case Report	Decreased lacrimation	N/A	Chronic right-hip osteomyelitis
Hilz et al., 1999 [20]	1	10.00	0	1	USA	Neurophysiologic study	Corneal scar	N/A	7 patients had burns and bone fractures, 5 had joint deformities of the knees and ankles
Yagev et al., 1999 [21]	15	3.75	8	7	Israel	Prospective case series	Corneal opacities, corneal ulcers	Visual acuity, refraction where possible, ex amination of corneal sensation, ocular movement examination, and a biomicroscopic examination of the anterior and posterior segments, tear-film breakup time test. Examination showed clear lenses and normal fundi in all patients.	Microcephalus arthrogryposis, deafness, congenital dislocation of the hip
Shatzky et al., 2000 [22]	12	N/A	0	12	Israel	Neurophysiologic study	Neurotrophic keratitis, corneal ulcers	N/A	Amputations of fingers and limbs, septic arthritis, Charcot joints
Jarade et al., 2001 [23]	1	6.00	0	1	Saudi Arabia	Case report	Corneal ulcers, eye redness	Schirmer reflex test, corneal reflex test, corneal scrapings from both eyes were subjected to Giemsa and Gram stains and cultured for bacteria and fungi	N/A
Shorer et al., 2001 [24]	7	4.90	0	7	Israel	Neurophysiologic study	Corneal ulcers	Visual acuity, evaluation of corneal sensation, ocular movement, biomicroscopic ex amination of the anterior and posterior segments, lacrimation was assessed by a tear-film breakup time test	N/A
Hiura et al., 2002 [25]	1	0.25	0	1	Japan	Case report	Blepharoptosis	N/A	High fever, deciduous teeth germ
Levy et al., 2004 [26]	1	2.00	0	1	Israel	Case report	Corneal erosion	N/A	Orbital cellulitis
Schalka et al., 2006 [27]	1	1.33	1	0	Brazil	Case report	Corneal ulcer	N/A	Oral lesions, unexplained recurrent fever episodes, osteomyelitis, bites fingers, self-mutilated
Amano et al., 2006 [28]	18	10.50	7	11	Japan	Prospective case series	Corneal opacities, Superficial punctate keratopathy (SPK), ciliary entropion, exotropia, esotropia, keratoconus	visual acuity, refraction by skiascopy or autorefractometer, slit-lamp examination of the anterior segment, tear breakup time (TBUT), Schirmer 1 test	N/A
John et al., 2010 [13]	1	3.00	1	0	India	Case report	Neurotrophic keratitis (watering, epithelial defect and hypopyon, corneal scar with thinning and vascularization)	Slit-lamp examination, corneal sensation checked with the tip of cotton wool, corneal scraping for bacterial and fungal smear (culture did not show any pathogens)	Autoamputation of fingers and toes and Charcot joints
Kucukdurmaz et al., 2012 [29]	1	10.00	0	1	Turkey	Case report	Corneal opacities	N/A	high fever, septic arthritis, osteo myelitis
Gao et al., 2013 [30]	1	6.00	0	1	China	Case report	Corneal ulcer, congenital keratitis	N/A	congenital nasal defects, submucous cleft palate and alveolar bone loss of the maxilla and mandible
Rapp et al. 2013 [31]	2	4.00	0	2	Germany	Case report	Corneal ulcer	N/A	Osteomyelitis, avascular necrosis
Iftikhar et al. 2013 [32]	1	30.00	1	0	Pakistan	Case report	Corneal opacity, sluggishly reactive pupils	Schirmer’s test	N/A
Guven et al., 2014 [33]	2	17.00	0	2	Turkey	Case report	Corneal opacities	N/A	N/A
Ofluoglu et al., 2015 [34]	1	11.00	0	1	Turkey	Case report	Corneal ulcer, scleral hyperemia, neurotrophic keratitis	N/A	Tissue loss due to burn injury of the second right finger, deep oral ulcers
Othman et al., 2016 [35]	1	2.00	1	0	Sudan	Prospective case series	Corneal scar	N/A	Dry hot skin, lower respiratory tract infection, hypotonia, insensitivity to pain
Altassan et al., 2017 [36]	2	4.00	1	1	Saudi Arabia	Case report	Corneal opacities	N/A	Dextrocardia, bilateral conductive hearing loss, Charcot deformity, autonomic dysfunction
Geng et al., 2018 [37]	5	7.92	0	0	China	Case series	Blepharoptosis	N/A	Damaged tongue, slow wound healing, fractures, osteomyelitis
Suresh et al., 2018 [38]	1	0.83	0	1	India	Case report	Recurrent corneal ulcer, neurotrophic keratitis	N/A	recurrent respiratory infections, several febrile episodes
Masri et al., 2019 [39]	4	1.00	1	3	Jordan	Retrospective analysis	Corneal ulcers	N/A	1 patient had hip joint dislocation and jaw fracture, 1 had bilateral hip and shoulder dislocation following a seizure, 1 had arthritis and fracture of upper limb, 1 had chronic osteomyelitis and acute transient renal failure, and all had microcephaly
López-Cortés et al., 2020 [40]	1	9.00	1	0	Ecuador	Case report	Corneal ulcers	N/A	Pneumonia, osteomyelitis, tibial fracture, osteochondroma, femoral fracture
Sethi et al., 2020 [41]	1	3.00	1	0	India	Case report	Neurotrophic keratitis, blurred vision, redness, watering, superficial punctate keratitis, nebulo-macular corneal scar	Schirmer’s test, slit-lamp, tear film, visual acuity	N/A
Elsana et al., 2021 [10])	32	15.13	13	19	Israel	Retrospective analysis	Corneal ulceration, corneal opacities, Superficial punctate keratitis, esotropia, exotropia blepharoptosis, astigmatism, keratoconus with corneal hy drops, decreased lacrimation	visual acuity, cycloplegic refraction, corneal sensitivity, TBUT, Schirmer test and posterior segment findings	N/A

^a^ Age is presented as the mean, N/A: not applicable.

**Table 2:** Distribution of ocular manifestations among patients with CIPA.

Ocular Manifestations	Sample Size (Cases)	Sample Size (Eyes)	Bilateral Involvement (Number of Cases)	References
Absent corneal sensitivity	56 (47.5%)	112	56 (100%)	[10,13,16,17,18,21,27,28,29,31,33,34,35,36,37,38,40]
Corneal ulcer	46 (38.98%)	54	8 (17.08%)	[10,21,22,23,26,27,30,31,34,38,39,40]
Corneal opacity	32 (27.11%)	51	19 (57.57%)	[10,16,18,21,28,29,32,33,36]
Decreased lacrimation	26 (22%)	52	N/A	[10,16,17,19,20,28,31,32,33,41]
Superficial punctate keratopathy	19 (16.1%)	27	N/A	[10,28,41]
Blepharoptosis	8 (6.7%)	9	1 (12.5%)	[10,17,25,37]
Neurotrophic keratitis	4 (3.3%)	5	3 (75%)	[13,22,34,41]
Exotropia	4 (3.3%)	4	N/A	[10,28]
Esotropia	4 (3.3%)	4	0	[10,28]
Corneal scar	4 (3.3%)		2 (50%)	[13,20]
Red eye	3 (2.5%)	6	3 (100%)	[23,34,41]
Ciliary entropion	2 (1.6%)	3	1 (50%)	[28]
Keratoconus	2 (1.6%)	4	2 (100%)	[10,28]
Epiphora	2 (1.6%)	4	2 (50%)	[13,41]
Astigmatism	2 (1.6%)	2	0	[10]
Congenital keratitis	1 (0.8%)	1	0	[30]
Hypopyon	1 (0.8%)	1	0	[13]
Sluggishly reactive pupil	1 (0.8%)	1	0	[32]
Blurred vision	1 (0.8%)	1	0	[41]
Tortuous retinal arteries	1 (0.8%)	1	0	[17]

**Table 3:** Association between reported mutations and ocular manifestations.

Ocular Manifestation	Mutation	Reference	Number of Cases
Corneal ulcers	rs763758904 (Arg602*)	López-Cortés 2020 [40]	1
	c.1860_186 1insT; p.Pro621fs	Masri 2019 [39]	4
	c.1561T-C in exon 13 and c.2057 G-A in exon 15	Gao 2013 [30]	1
	“TrkA: 1926-ins-T” and “TrkA: Pro-689-Leu”	Shatzky 2000 [22]	12
	TrkA: 1926-ins-T	Shorer 2001 [24]	7
	homozygous c.274dupG, pGlu92GlysfsX81	Rapp 2013 [31]	1
	TrkA: 1926-ins-T	Elsana 2021 [10]	8
	frameshift mutation NTRK1 c.717delG and pMet239fs	Suresh 2018 [38]	1
Corneal opacity	three missense mutations (p.Arg110Asp, p.Arg643Gln, p.Leu694- Pro) and two nonsense mutations (p.Ser146Ter, p.Lys476Ter)	Altassann 2017 [36]	2
	homozygous c.2001C-T alteration in exon 15	Guven 2014 [33]	2
Absent corneal reflex	five missense mutations (c.1784T>G, c.1927C>T, c.2056C>T, c.2152G > A, and c.2293C>T), one nonsense mutation (c.1786C>T, R596*), two frameshift mutations (c.963delG, c.1736delT), and four intronic splicing mutations (c.851-33T>A, c.287+2dupT c.850+1G>A, c.2188-11G>A)	Geng 2018 [37]	5
	rs763758904 (Arg602*)	López-Cortés 2020 [40]	1
	three missense mutations (p.Arg110Asp, p.Arg643Gln, p.Leu694- Pro) and two nonsense mutations (p.Ser146Ter, p.Lys476Ter)	Altassann 2017 [36]	2
	homozygous c.2001C-T alteration in exon 15	Guven 2014 [33]	2
	homozygous c.274dupG, pGlu92GlysfsX81	Rapp 2013 [31]	2
	TrkA: 1926-ins-T	Elsana 2021 [10]	13
	frameshift mutation NTRK1 c.717delG and pMet239fs	Suresh 2018 [38]	1
Decreased Lacrimation	TrkA: 1926-ins-T	Elsana 2021 [10]	9
	homozygous c.274dupG, pGlu92GlysfsX81	Rapp 2013 [31]	2
	homozygous c.2001C-T alteration in exon 15	Guven 2014 [33]	2
Superficial Punctate Keratopathy	TrkA: 1926-ins-T	Elsana 2021 [10]	6
blepharoptosis	TrkA: 1726-del-C	Hiura et al. [25]	1

## Data Availability

The data supporting the findings of this study are openly available in the Supplementary Material.

## Supplementary Materials

The following supporting information can be downloaded at: https://www.mdpi.com/article/10.3390/vision9030062/s1, Table S1: This excel sheet comprises the details for the included studies.
